# LINC01088 promotes the growth and invasion of glioma cells through regulating small nuclear ribonucleoprotein polypeptide A transcription

**DOI:** 10.1080/21655979.2022.2051786

**Published:** 2022-04-07

**Authors:** Tao Peng, Dong-Liang Chen, Shi-Lan Chen

**Affiliations:** Department of Neurosurgery, The First People’s Hospital of Qinzhou/The Tenth Affiliated Hospital of Guangxi Medical University, Qinzhou, Guangxi, China

**Keywords:** LncRNA, glioma, LINC01088, SNRPA, transcription, invasion

## Abstract

Altered long non-coding RNAs (LncRNAs) exert pivotal parts in pathogenic processes in glioma. Here, we uncovered a differentially expressed long intergenic non-coding RNA 1088 (LINC01088) in glioma and elucidated the molecular mechanism by which LINC01088 affected the malignant phenotypes of glioma cells. Functionally, LINC01088 silencing degraded cell proliferation, invasion in glioma, while LINC01088 overexpression elicited opposite results. Mechanistically, we verified LINC01088 physically interacted with small nuclear ribonucleoprotein polypeptide A (SNRPA) and regulated the expression of SNRPA at the transcription level. Phenotypic analysis ascertained that LINC01088 substantively aggravated glioma cell progression in an SNRPA-dependent manner, and SNRPA played a pivotal part in the tumor-promoting properties of LINC01088. Our findings revealed a novel mechanism by which LINC01088 exerted pro-oncogenic functions through binding with SNRPA and transcriptionally regulating SNRPA mRNA in glioma.

## Graphical abstract

**Figure:**
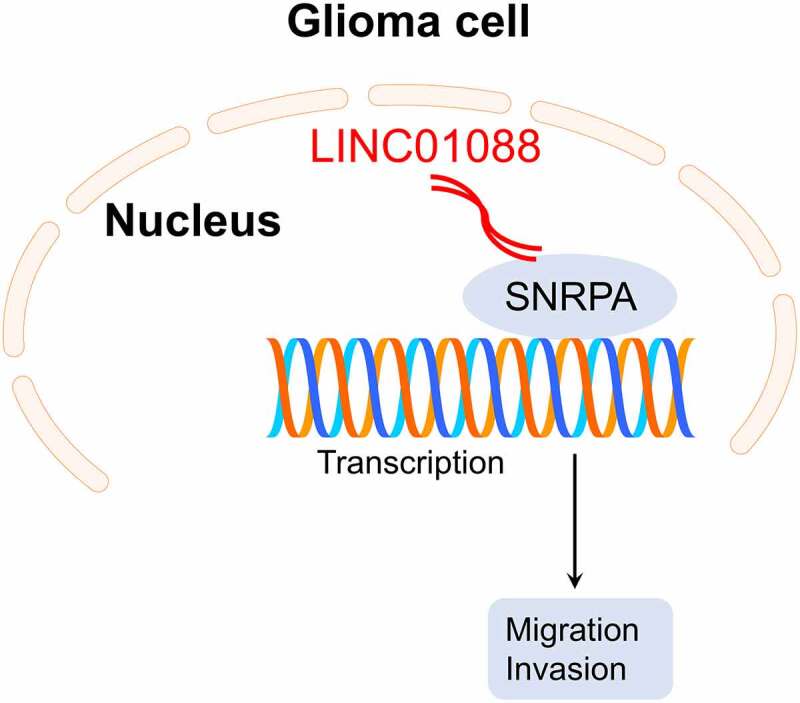

## Introduction

Glioma remains the prevalent primary intracranial tumor in the brain [1]. Currently, standard glioma therapies include surgical removal of the primary lesion and chemoradiotherapy [2]. Apart from genetic heterogeneity, rapid growth, extensive aggressive behavior, and therapeutic resistance of glioma, the adverse prognosis for glioma patients is partially attributed to insufficient understanding of complicated mechanisms driving glioma progression [3]. It is thus imperative to delineate the precise mechanisms involved in glioma progression and further identify robust biomarkers.

Dysregulated transcripts including LncRNAs can be the primary characteristic in cancers [4,5]. LncRNAs, a subset of ncRNAs characterized by >200 nucleotides in length, are identified to be implicated in multiple biological processes in malignancies [6,7]. For example, an extensive body of evidence has illuminated that lncRNAs serve as competing endogenous RNAs (ceRNAs) to affect the deterioration of cancer [8,9]. LncRNAs also mediate gene expressions at transcriptional or posttranscriptional level [10].

The previous report proved that LINC01088 restrains the oncogenesis of ovarian epithelial cells *via* modulating microRNA-24-1-5p (miR-24-1-5p) [11]. In human non-small cell lung cancer (NSCLC), LINC01088 accelerates cell growth through scaffolding Enhancer Of Zeste 2 Polycomb Repressive Complex 2 Subunit (EZH2) and lessening p21 [12]. However, the specific role of LINC01088 in glioma remains poorly elucidated. In this study, with the assistance of RNA-seq, we identified that LINC01088 is highly expressed in glioma.

In the current study, we found that attenuation of LINC01088 expression impaired glioma cell proliferation, clonogenic activity, and the invasive phenotype *in vitro*. Also, LINC01088 knockdown inhibited the tumorigenic property of glioma cells *in vivo*. Further studies substantiated that LINC01088 interacted with SNRPA and SNRPA mainly mediated the tumor-promoting effect of LINC01088. This work indicates that LINC01088 acts as a tumor promoter in glioma and might be an emerging target against glioma.

## Materials and methods

### Cell culture

Human glioma cell lines SHG-44 and U87 were bought from Nanjing Cobioer Biotechnology Co., Ltd (Nanjing, China). A human microglia cell line HMC3 and glioma cell line U251 and T98G were purchased from Procell Life Science&Technology Co., Ltd (Wuhan, China). Glioma cells and HMC3 were maintained in Dulbecco’s Modified Eagle Medium (DMEM) (Thermo Fisher Scientific, MA, USA) supplemented with 10% fetal bovine serum (FBS) and cultured in a humidified 37°C, 5% CO_2_ incubator.

### Clinical samples preparation

Thirty-five pairs of serum specimens from patients with glioma and controls were collected from the First People’s Hospital of Qinzhou. All cases were diagnosed with glioma by two independent pathologists. Informed written consent was signed by all participants. The clinicopathological data are summarized in Table 1. Our study protocol was approved by the Ethics Committee of the First People’s Hospital of Qinzhou (No. [2017]1008) in compliance with the Declaration of Helsinki. The ethical approvement can be found in Supplementary Material. Serum samples were snap-frozen in liquid nitrogen and stored at −80°C before RNA isolation.

**Table 1.** The clinicopathologic features in patients with glioma

Clinical parameter	Expression of LINC01088		P value	Expression of SNRPA		P value
	High	Low		High	Low	
**Age (years)**			0.266			0.709
≤45	4	6		3	7	
>45	11	14		14	11	
**Gender**			0.71			0.685
Female	8	13		11	10	
Male	7	7		6	8	
**Location**			0.517			0.210
Frontal	10	9		8	11	
Parietal	2	3		4	1	
Occipital	1	7		3	5	
Temporal	2	1		2	1	
**WHO grade**			<0.01			<0.01
Low grade (I~ II)	2	12		3	11	
High grade (III~IV)	13	8		14	7	

### Cell transfections

U251 or T98G cells (3 × 10^3^) were seeded in six-well plates 24 h before transfection. U251 or T98G cells were transduced with short hairpin RNA against LINC01088 (sh-LINC01088 #1: CCGGGAAGGTTCACATGCCTCAAGGCTCGAGCCTTGAGGCATGTGAACCTTCTTTTTG or #2: CCGGGGATCTCTCTCTCTCAGATTTCTCGAGAAATCTGAGAGAGAGAGATCCTTTTTG) or shRNA-targeting SNRPA (sh-SNRPA #1: CCGGCTTTAAGATCACGCAGAACAACTCGAGTTGTTCTGCGTGATCTTAAAGTTTTTG or #2: CCGGCCTCAATGAGAAGATCAAGAACTCGAGTTCTTGATCTTCTCATTGAGGTTTTTG) using a Lipofectamine 3000 kit (Thermo Fisher Scientific) to reduce the level of LINC01088 and SNRPA level, respectively. The full-length LINC01088 or SNRPA cDNA was cloned into pcDNA3.1 (Sangon Biotech Co., Ltd, Shanghai, China). LINC01088 overexpression vector (oe-LINC01088) or SNRPA overexpression vector (oe-SNRPA) was transfected into cells to raise their expression levels. The corresponding negative control (sh-NC) or empty vector (vector) served as a negative control. All plasmids and vectors were provided by GeneChem Biotech (Shanghai, China). Transfected cells were analyzed 48 hours post-transfection. In stable transfection, sh-LINC01088 was cloned into lentiviral vector pLKO.1 (Sangon). Production of lentiviral particles was conducted according to the manufacturer’s protocol. T98G cells were transfected with lentiviral constructs for 48 h. Stable cell lines were selected using 1 mg/mL puromycin for 1-week [13,14].

### Transwell invasion

Invasive capacity was measured using a Transwell chamber (24-well) with 8 μm polycarbonate filters coated with Matrigel. Two hundred microliters of cell suspension (1000 cells per well) in an FBS-free medium were plated into the upper chamber. The lower chamber was filled with 600 μL of medium supplemented with 10% FBS. 24 h post-incubation, the invaded cells underneath the membrane were dyed with 1% crystal violet (Beyotime) for 15 min, and the number of invaded cells was counted.

### Cell growth

After transfected, U251 or T98G cells were cultured into 96 well microplates (3 × 10^3^). After 1, 2, 3, 4, or 5 days, 10 μL of Cell counting Kit-8 (CCK-8) solution (Beyotime) was then added to plates. Following 2 h of incubation, the optical density (OD) was detected with a microplate reader at 450 nm. In clone formation assay, cells (1000 per well) were cultured in 6 well plates to form colonies. Fourteen days post-growth, the colonies were dyed with crystal violet. Colonies containing more than 50 cells were counted.

### Subcellular localization

Cy3-labeled probe for LINC01088 was synthesized by Sangon Biotech Co., Ltd, fluorescence in situ hybridization (FISH) assay was conducted using a Ribo™ Fluorescent In Situ Hybridization kit (RiboBio, Guangzhou, China) according to the instruction manuals.

### qRT-PCR assay

Total RNA in cells or serum samples was extracted by using TRIzol reagent (Beyotime) and reverse transcribed to cDNA with a PrimeScript RT Reagent Kit (TaKaRa). For the isolation of nuclear and cytoplasmic RNA, nuclear, cytoplasmic and total RNA were extracted using a PARIS™ kit (Thermo Fisher Scientific). qRT-PCR was implemented using an SYBR Green RT-PCR kit (Bio-Rad, Hercules, CA, USA) on a CFX96 RT-PCR Detection System (Bio-Rad). The PCR primers were designed by Sangon Biotech. The sequences of primers are as follows: LINC01088 forward: 5’-CTTCCCTTCCTCCATGATT-3’ reverse: 5’-ATGTGGGGACATTGATGCTT-3’; GAPDH forward 5’-TGTGGGCATCAATGGATTTGG-3’ reverse 5’-ACACCATGTATTCCGGGTCAAT-3’; SNRPA forward 5’-ACCCGCCCTAACCACACTATT-3’ reverse 5’-GAGAAGATGGCGTACAGGGAC-3’. GAPDH was used as the internal reference. Relative expression of genes was calculated by means of 2^−ΔΔCt^.

### Immunoblotting assay

Total proteins were prepared from cells using RIPA buffer. Equal amounts of proteins were separated using 10% SDS-PAGE and immediately transferred to a PVDF membrane (Millipore). These blots were incubated with primary anti-SNRPA (sc-376,027, Santa Cruz, CA, USA) or anti-GAPDH (sc-47,724, Santa Cruz, CA, USA) at 4°C overnight followed by incubation with HRP-labeled secondary antibody (Beyotime). The signals were visualized using enhanced chemiluminescence (Millipore).

### RNA pull-down assay

RNA pull-down assay was performed using a Pierce Magnetic RNA-Protein Pull-Down Kit (Thermo Fisher Scientific). The full-length LINC01088 sequence was amplified by using a MEGAscript™ T7 Transcription Kit (Thermo Fisher Scientific) and was biotinylated with Pierce RNA 3′ End Desthiobiotinylation Kit (Thermo Fisher Scientific). Biotin-labeled LINC01088 was captured by the streptavidin magnetic beads and then incubated with T98G or U251 cell lysates at 4°C for 6 h. Subsequently, the mixture was washed twice, and the pulled-down complexes were verified by means of western blot.

### RNA immunoprecipitation (RIP)

The interaction between SNRPA and LINC01088 was determined using a Magna RIP RNA-binding protein (RBP) immunoprecipitation kit (Millipore). Total RNA from cells was precipitated with anti-SNRPA or IgG antibody (Santa Cruz Biotechnology). The RNA-protein binding compound was absorbed with magnetic beads and qRT-PCR was utilized to determine the enrichment of immunoprecipitated LINC01088.

### Glioma cell growth in vivo

Hundred microliters of T98G cells (5 × 10^6^) stably transfected with sh-Con or sh-LINC01088 in PBS were injected subcutaneously in the dorsal flank of Balb/c nude mice (n = 3 in each group). Tumor volume = length × width^2^/2. Twenty-eighth day after inoculation, mice were sacrificed by cervical dislocation. Animal ethics approval was granted by the Animal Ethics Committee of the First People’s Hospital of Qinzhou (No. [2019]316). Excised tumor tissues were subjected to immunohistochemistry (IHC) staining as previously described [15].

### Statistical analysis

Data are shown as mean ± SD, and statistical analysis is completed using GraphPad Prism 8.0. Differences were analyzed using a t-test or one-way analysis of variance (ANOVA). Analyses of association between LINC01088 or SNRPA and clinicopathological features of patients with glioma were performed using the Mann-Whitney U-test. Pearson’s correlation test was used to determine the correlation between LINC01088 and SNRPA. *P* < 0.05 was statistical significance.

## Results

This study aimed to reveal the expression level, biological significance, and potential mechanism of LINC01088 in glioma. We demonstrated that LINC01088 was upregulated in glioma tissues and cell lines. Mechanistically, LINC01088 exerted the pro-oncogenic functions through binding with SNRPA and transcriptionally regulating SNRPA mRNA in glioma. Overall, this study verified the function and mechanism of the LINC01088/SNRPA regulatory axis in glioma.

### The expression of LINC0108 in glioma

**Figure 1.**
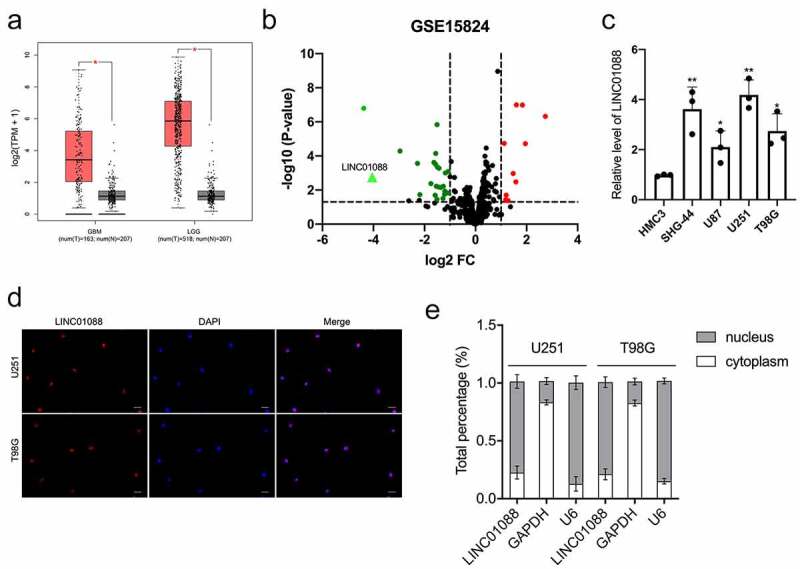
**The expression profile, cellular location, and distribution of LINC01088**. (a) GEPIA (http://gepia.cancer-pku.cn/index.html) results of up-expression of LINC01088 in glioblastoma multiforme (GBM) and brain lower-grade glioma (LGG) tissues. (b) The volcano map of differentially expressed lncRNAs in GSE15824. (c) qRT-PCR analysis of LINC01088 expression in microglia cell line HMC3 and glioma cell lines (U251, T98G, SHG-44 and U87). (d) The expression and location of LINC01088 (green) in U251 and T98G cells was determined by FISH assay with confocal microscopy. DAPI-stained nuclei were blue. Scar bar: 50 μM. (e) The expression level of LINC01088 in the subcellular fractions of U251 and T98G cells was detected by qRT-PCR. All data were presented as means ± SD of three independent experiments. Values are significant at *P < 0.5 and **P < 0.01 as indicated.

The differential expression of LINC01088 in glioma was detected *via* the Gene Expression Profiling Interactive Analysis (GEPIA) website (http://gepia.cancer-pku.cn/index.html), we observed that the level of LINC01088 was tremendously elevated in glioblastoma multiforme (GBM) and brain lower-grade glioma (LGG) tissues in comparison to nontumor samples (Figure 1a) [16]. The differentially expressed lncRNAs associated with glioma were further analyzed using the microarray dataset GSE15824 and shown in the volcano plot. When compared with normal brain tissue, red dots represent down-regulated lncRNAs and green dots represent up-regulated lncRNAs in glioma. We noticed that the LINC01088 level in glioma tissue was higher than in normal tissue (Figure 1b). Moreover, we tested the endogenous expression level of LINC01088 in glioma cell lines, and the level of LINC01088 was remarkedly raised in glioma cells when compared with HMC3 (Figure 1c). To identify cellular localization of LINC01088, FISH confirmed the distribution of LINC01088 in glioma cells (Figure 1d). qRT-PCR analysis of nuclear and cytoplasmic RNAs displayed that LINC01088 was primarily localized in the nucleus (figure 1f). All these data indicated that LINC01088 expression is elevated in glioma.

### Silencing of LINC01088 suppresses glioma cell growth and invasion

**Figure 2.**
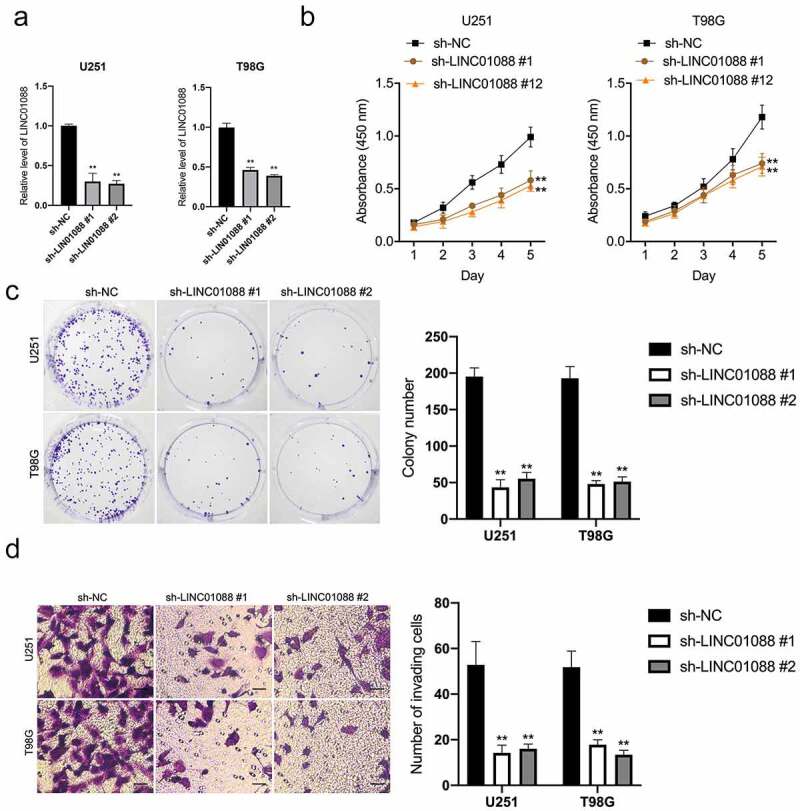
**Knockdown of LINC01088 inhibits proliferation, colony formation and invasion of glioma cells**. (a) The inhibiting efficiency of different shRNA-LINC01088 (sh-LINC01088 #1, #2) was evaluated in U251 and T98G cells respectively. (b) U251 and T98G cells were transfected with sh-LINC01088 or negative control (sh-NC), and the proliferative ability was assessed by CCK-8 assay. (c) Colony formation assay was performed in transfected cells for 2 weeks. Representative images (left) and relative cells number of colonies (right) are shown. (d) Transwell assay was applied to assess the invasion ability of those transfected cells. The histogram shows the percentage of invaded cells. Scar bar: 50 μM. *P < 0.5 and **P < 0.01 compared with sh-NC.

To explore the functional roles of LINC01088, two short interfering RNAs against LINC01088 (sh-LIN01088 #1, LINC01088 #2) were transfected into T98G and U251 cells (Figure 2a). sh-LINC01088 #2 was applied for subsequent experiments because it had a higher silencing efficiency. CCK-8 and clonogenic assay exhibited that silencing of LINC01088 inhibited glioma cell proliferation and clone formation (Figure 2b-c). In addition, transwell assay suggested that LINC01088 inhibition drastically attenuated cell invasion capacity (Figure 2d). In contrast to knock-down, enhancing LINC01088 expression heightened cell growth, clone formation, and invasive capacity in U251 cells (Supplemental Figure 1A-D).

### LINC01088 is associated with SNRPA

**Figure 3.**
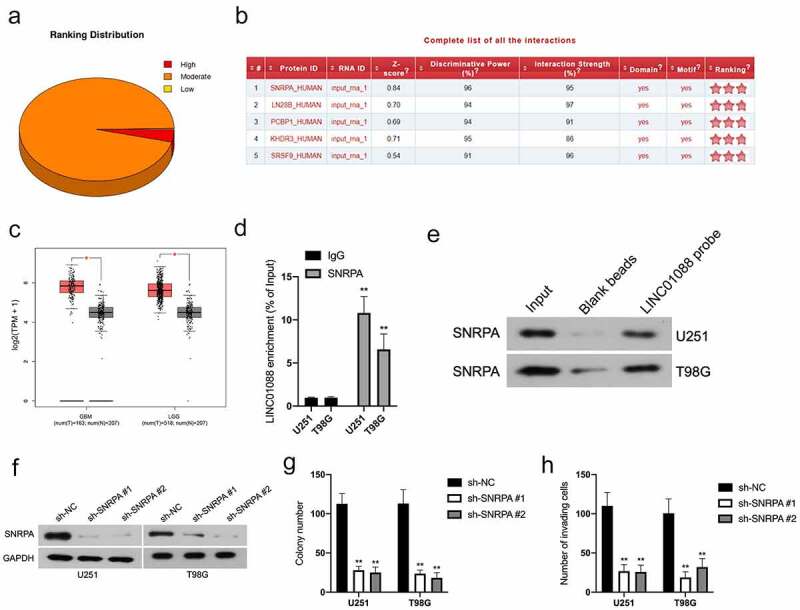
**The interaction between LINC01088 and SNRPA**. (a) The catRAPID algorithm showed the putative association of LINC01088 with RNA binding proteins (RBPs). (b) The analysis of catRAPID algorithm showed the ranking distribution of protein that latent-binding to LINC01088. SNRPA possessed a higher binding capacity. (c) GEPIA (http://gepia.cancer-pku.cn/index.html) results of up-expression of SNRPA in GBM and LGG tissues. (d) RIP experiment was performed by using the SNRPA and IgG antibodies, and the level of the co-precipitated RNAs was determined by using qRT-PCR. (e) Total protein was extracted from U251 and T98G cells and utilized in a biotinylated LINC01088 pull-down assay. Proteins associated with LINC01088 were explored by western-blot with anti-SNRPA antibody. F, U251 and T98G cells were transfected with sh-SNRPA or sh-NC, and the expression of SNRPA was assessed by immunoblotting. (g) Colony formation analysis of glioma cells growth. H, Transwell analysis of glioma cells invasion. **P < 0.01 compared with sh-NC.

Emerging studies have reported that lncRNAs interact with DNA or RNA binding proteins (RBPs) and finally promote tumor progression. To unravel the molecular mechanism by LINC01088 in glioma, the catRAPID algorithm was utilized to seek the potential RBPs of LINC01088. Among those RBPs, SNRPA possesses the highest interaction strength with LINC01088 (Figure 3a-b). With the aid of TCGA, we found that GBM and LGG tissues have a higher level of SNRPA level when compared to nontumor samples (Figure 3c). Subsequently, RIP assay using the lysates of U251 and T98G cells was implemented, and LINC01088 was tremendously enriched by anti-SNRPA antibody (Figure 3d). Next, the RNA pull-down assay combination western blot was performed with the biotin-labeled LINC01088. As illustrated in Figure 3e, SNRPA could be pulled down by biotin-labeled LINC01088. Finally, endogenous SNRPA expression in U251 and T98G cells was effectively knocked down by sh-SNRPA (Figure 3f). Analogous to LINC01088 inhibition, attenuation of SNRPA expression had a suppressive effect in glioma cells clonogenic and invasive phenotype (Figure 3g-h).

### The mRNA level of SNRPA is regulated by LINC01088

**Figure 4.**
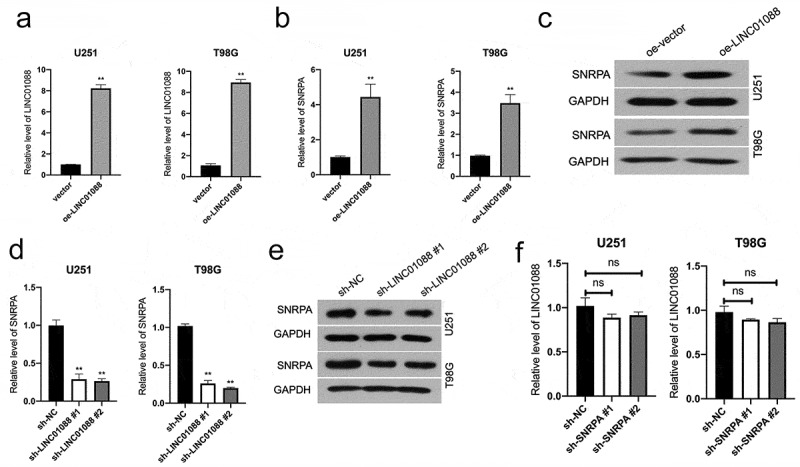
**Effect of LINC01088 on SNRPA expression**. (a-c), The mRNA level and protein expression of SNRPA were evaluated in U251 and T98G cells after transfected with oe-LINC01088 plasmid by qRT-PCR or western blot, respectively. **P < 0.01 compared with vector. (d-e), The mRNA level and protein expression of SNRPA were evaluated in U251 and T98G cells after transfected with sh-LINC01088 plasmid by qRT-PCR or western blot, respectively. (e) The expression of LINC01088 in indicated cells was detected using qRT-PCR. **P < 0.01 compared with sh-NC.

To elucidate the biological effect of LINC01088-SNRPA interactions and examine whether LINC01088 affects SNRPA expression, we transiently transfected a LINC01088-expression vector (oe-LINC01088) into U251 and T98G cells (Figure 4a). We found that upregulation of LINC01088 boosted the mRNA level and protein expression of SNRPA (Figure 4b-c). Furthermore, LINC01088 knockdown in U251 and T98G cells decreased SNRPA mRNA levels and protein expressions (Figure 4d-e). While any change in LINC01088 level was not detected upon SNRPA knockdown in glioma cells (Figure 4f).

### SNRPA is critical for LINC01088 to exert promotion function

**Figure 5.**
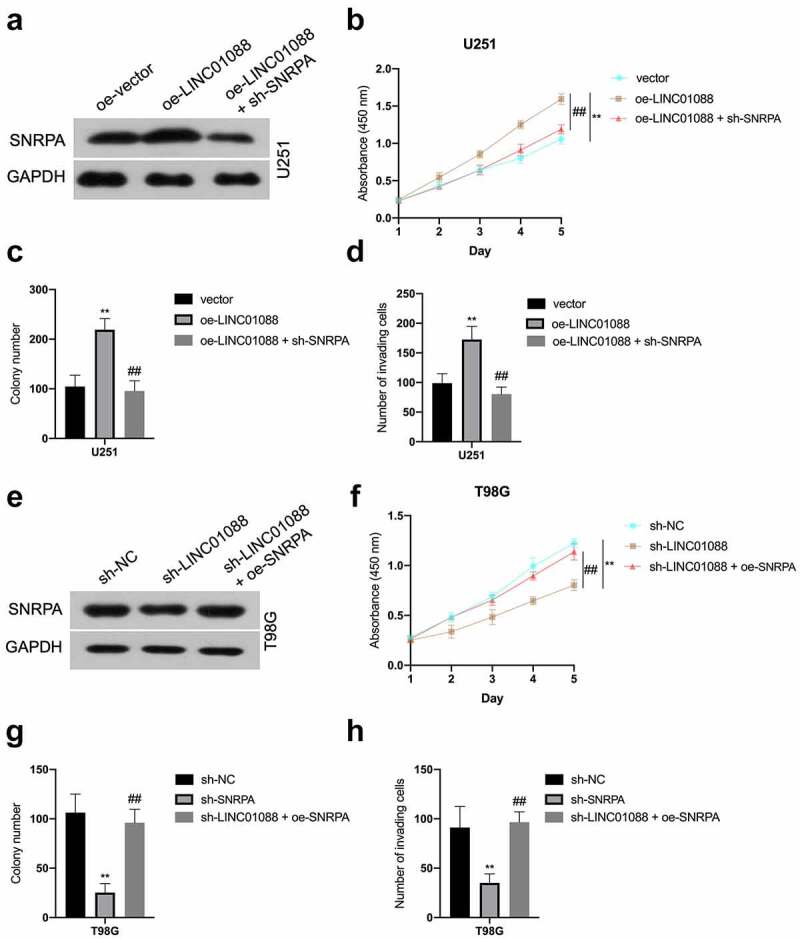
**SNRPA mediates the function of LINC01088 to promote the proliferation and invasion of glioma cells**. (a) The protein expression of SNRPA was measured using western blot when SNRPA was knocked down in U251 cells transduced with oe-LINC01088 plasmid. (b) The growth curve was detected by CCK-8 assay in U251 cells. (c-d) The colonizing ability and invasion of cotransfected U251 cells were detected by colony formation assay or Transwell, respectively. **P < 0.01 compared with vector, ^##^*P* < 0.01 compared with oe-LINC01088. (e) The protein expression of SNRPA was measured using western blot when SNRPA was rescued in T98G cells transduced with sh-LINC01088 plasmid. (f) The growth curve was detected by CCK-8 assay T98G cells. (g-h), The colonizing ability and invasion of cotransfected T98G cells were detected by colony formation assay or Transwell, respectively. **P < 0.01 compared with sh-NC, ^##^*P* < 0.01 compared with sh-SNRPA.

Since SNRPA expression is regulated by LINC01088 in a transcriptional manner, we presumed SNRPA might be a critical element for LINC01088 in glioma progression. Coincidently, co-transfection of sh-SNRPA abated the LINC01088-upregulated SNRPA mRNA level and expression in U251 cells (Figure 5a and Supplemental Figure 2A-B), as well as cell proliferation, colony-forming, and invasive potency in U251 cells (Figure 5b-d). Conversely, SNRPA overexpression rescued sh-LINC01088-decreased SNRPA level in T98G (Figure 5e), as well as T98G cells proliferation and aggressive phenotype, could be rescued by upregulation of SNRPA (Figure 5f-h). In sum, these observations disclosed that SNRPA is an essential downstream factor for LINC01088 exerting cancer-promoting actions in glioma.

### Depletion of LINC01088 inhibits the tumorigenicity of glioma cell

**Figure 6.**
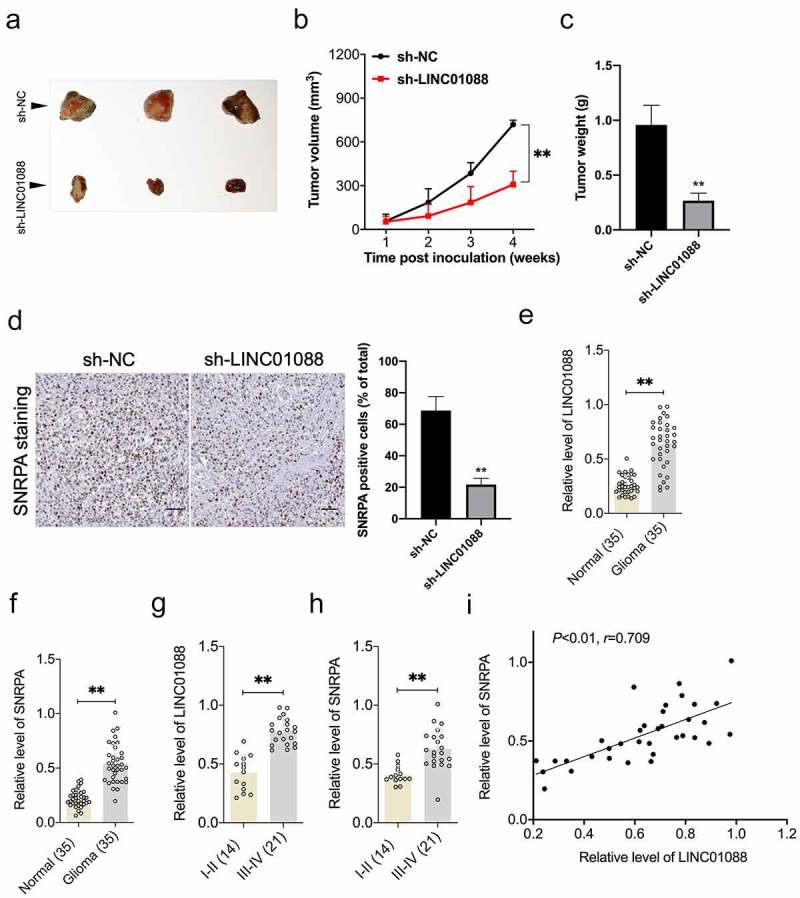
**Knockdown of LINC00460 inhibits glioma cell growth *in vivo***. (a) Representative images of tumor-bearing nude mice inoculated with T98G cells stably transduced with sh-NC or sh-LINC01088. (b-c) The volumes and weights of tumors from tumor-bearing nude mice treated with sh-NC or sh-LINC01088 transduced T98G cells. (d) The level of SNRPA expression in tumor tissues was determined by IHC staining. Scar bar: 50 μM. (e) Expression level of LINC01088 was identified in 35 paired serum specimens from glioma patients and control normal by qRT-PCR. (f) Expression level of SNRPA in serum specimens from glioma patients or control normal. (g) qRT-PCR analysis of the relative expression of LINC01088 in patients with low-grade glioma (14) and high-grade glioma (21). (h) qRT-PCR analysis of the relative expression of SNRPA in patients with low-grade glioma (14) and high-grade glioma (21). (i) The Pearson correlation between LINC01088 level and SNRPA level was measured in the same set of glioma patients. Value is significant at **P < 0.01 as indicated.

To corroborate the role of LINC01088 in glioma cell growth, a sh-LINC01088 stable transfected T98G cell line was constructed. After cells were inoculated into nude mice, we observed that knockdown of LINC01088 substantially prohibited tumor growth in contrast to the sh-NC group (Figure 6a-c). The results from IHC staining further validated the inhibition in SNRPA expression caused by sh-LINC01088 *in vivo* (Figure 6d). Finally, the endogenous levels of LINC01088 and SNRPA were detected using qRT-PCR in 35 paired serum specimens from glioma patients and control normally. The results showed that LINC01088, as well as SNRPA level, were extremely higher in the glioma group than in normal (Figure 6e-f). Importantly, qRT-PCR also displayed that in high-grade glioma, the relative levels of LINC01088 and SNRPA were higher than in low-grade glioma (Figure 6g-h, p < 0.01). Furthermore, LINC01088 expression was positively correlated with SNRPA expression in serum specimens from patients with glioma (Figure 6i).

## Discussion

In the current study, we identified that the LINC01088 level is significantly elevated in glioma when compared with that in normal. Gain- and loss-of-function assays testified that LINC01088 impaired the growth and invasion of glioma cells *in vitro*. Consistently, attenuation of LINC01088 expression exhibited tumor-inhibition effect in glioma cells growth *in vivo*.

LncRNAs are frequently dysregulated in cancers and have been linked to prognosis in clinical [5,17]. Here, we preferentially covered that LINC01088 was tremendously elevated in glioma, and its high aberrant expression was positively associated with glioma grade. Either as an onco-lncRNA or a tumor-inhibitor, LINC01088 exhibits critical biological significance in cancers, such as lung cancer and ovarian carcinoma [11,12]. LncRNA exerts biological function through chromatin modification, transcriptional regulation, post-transcriptional regulation, or competing endogenous RNAs (ceRNA), etc. [18]. Several studies have already evaluated the role of lncRNA in regulating of different signaling pathways in different tumor cells [19,20]. Moreover, due to the different origins of each type of cancer and the microenvironments surrounding tumor cells, the mechanisms of each lncRNA are different [21]. However, the specific expression and functional implication of LINC01088 in glioma remain unreported. Our results for the first time manifested the biological meaning of LINC01088 and indicated that LINC01088 might serve as a candidate target for glioma.

Emerging studies have verified that lincRNAs associate with a range of biomolecules, including mRNAs, transcription factors (TFs), and RBPs [22,23]. We identified SNRPA as an RBP of LINC01088 in glioma cells. The RIP and RNA pull-down analysis suggested that LINC01088 physically interacted with SNRPA. As for tumor progression, an early report has manifested that SNRPA expression is abnormally elevated in hepatocellular carcinoma (HCC) [24]. The upregulation of SNRPA expression is observed in gastric carcinoma (GC) and SNRPA expression increasing or inhibition of SNRPA results in reinforced or repressed growth phenotype of GC cell [25]. Similarly, we demonstrated that SNRPA exerts its oncogene effect in glioma.

In rescue assay, after SNRPA was knocked down in LINC01088 over-expression cells or SNRPA overexpression in LINC01088-downexpressing cells, the growth and invasion trends were reversed. As a result, LINC01088 effectively induces glioma cell growth and invasion in an SNRPA-dependent manner, and SNRPA mediated the pro-tumorigenic efficacy of LINC01088. Our study also shown the LINC01088 level is positively correlated with SNRPA level in glioma. Nuclear genes bind to the promoter regions of distinct target genes, upregulate gene expression, and promote cancer cells metastasis. In spite of a study that has confirmed nerve growth factor (NGF) gene acts as a functional downstream effector of SNRPA and is essential for SNRPA modulated gastric cancer cells growth [25]. Whether SNRPA can bind directly to the NGF promoter region needs to be explored further. Similarly, the binding sites for LINC01088 and SNRPA are needed to be predicted using PRIdictor database and validated by Chromatin immunoprecipitation (ChIP) assay in follow-up work. Moreover, the number of clinical samples was too small to illuminate the prognosis significance of LINC01088 in glioma patients.

## Conclusion

In sum, we uncovered an upregulated lncRNA, LINC01088 in glioma. LINC01088 deletion inhibits glioma progression both *in vitro* and *in vivo*. LINC01088 physically binds SNRPA and SNRPA is implicated in the tumor-promoting properties of LINC01088 in glioma.

## Supplementary Material

Supplemental Material
