# Supplementary material for: LINC01088 promotes the growth and invasion of glioma cells through regulating small nuclear ribonucleoprotein polypeptide A transcription
Source: Bioengineered. 2022 Apr 7;13(4):9172–83. doi: 10.1080/21655979.2022.2051786 (PMC9162022; doi:10.1080/21655979.2022.2051786)

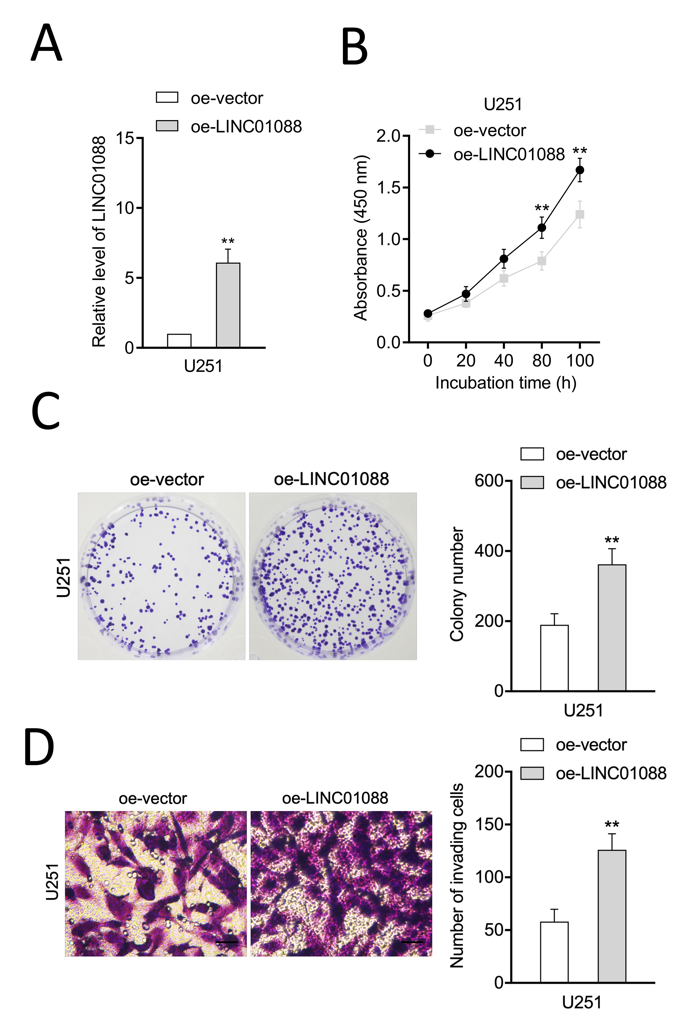


Supplemental Fig 1. Overexpression of LINC01088 enhances the proliferation, colony formation, and invasion of U251 cells. A, U251 cells were transfected with oe-vector or oe-LINC01088. The expression of LIC01088 was detected using qRT-PCR B, The proliferative ability was assessed by CCK-8 assay. C, Colony formation assay was performed in transfected cells for 2 weeks. Representative images (left) and relative cells number of colonies (right) are shown. D, Transwell assay was applied to assess the invasion ability of those transfected cells. The histogram shows the percentage of invaded cells. Scar bar: 50 μM. ^**^*P*<0.01 compared with oe-vector.


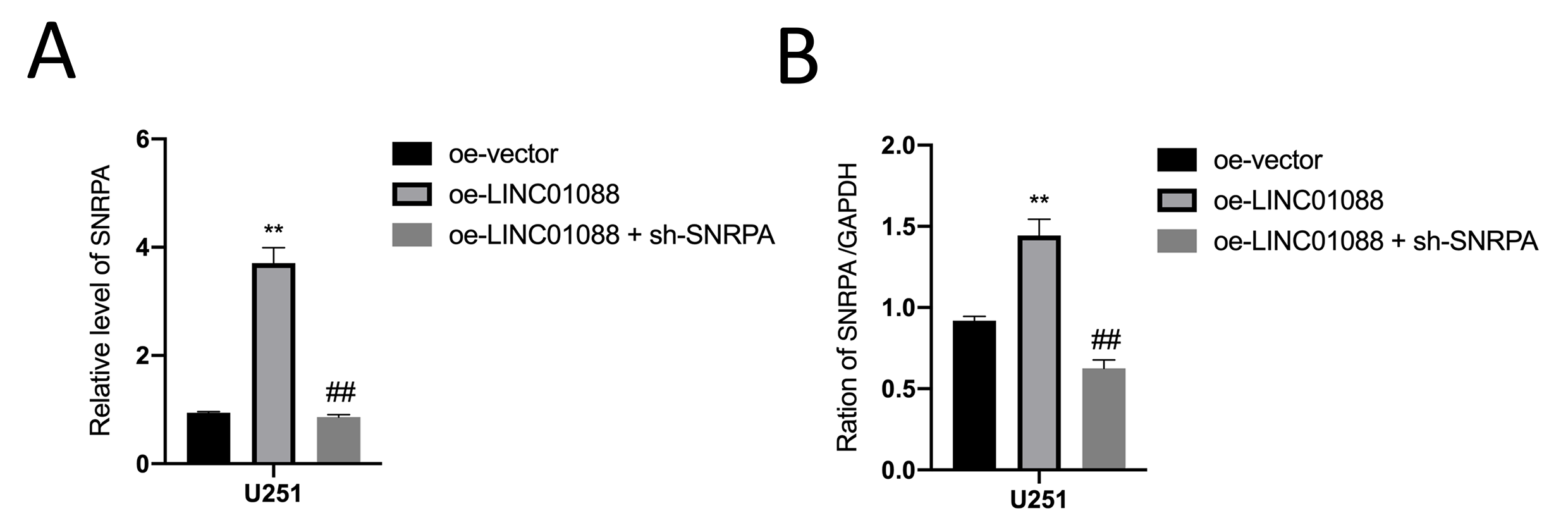


Supplemental Fig 2. The level of SNRPA is inhibited by sh-SNRPA in LINC01088 overexpressing U251 cells. A, The mRNA level of SNRPA was measured using qRT-PCR in U251 cells. B, Quantitative measurement of the protein expression level of SNRPA. ^**^*P*<0.01 compared with oe-vector, ^##^*P*<0.01 compared with oe-LINC01088.


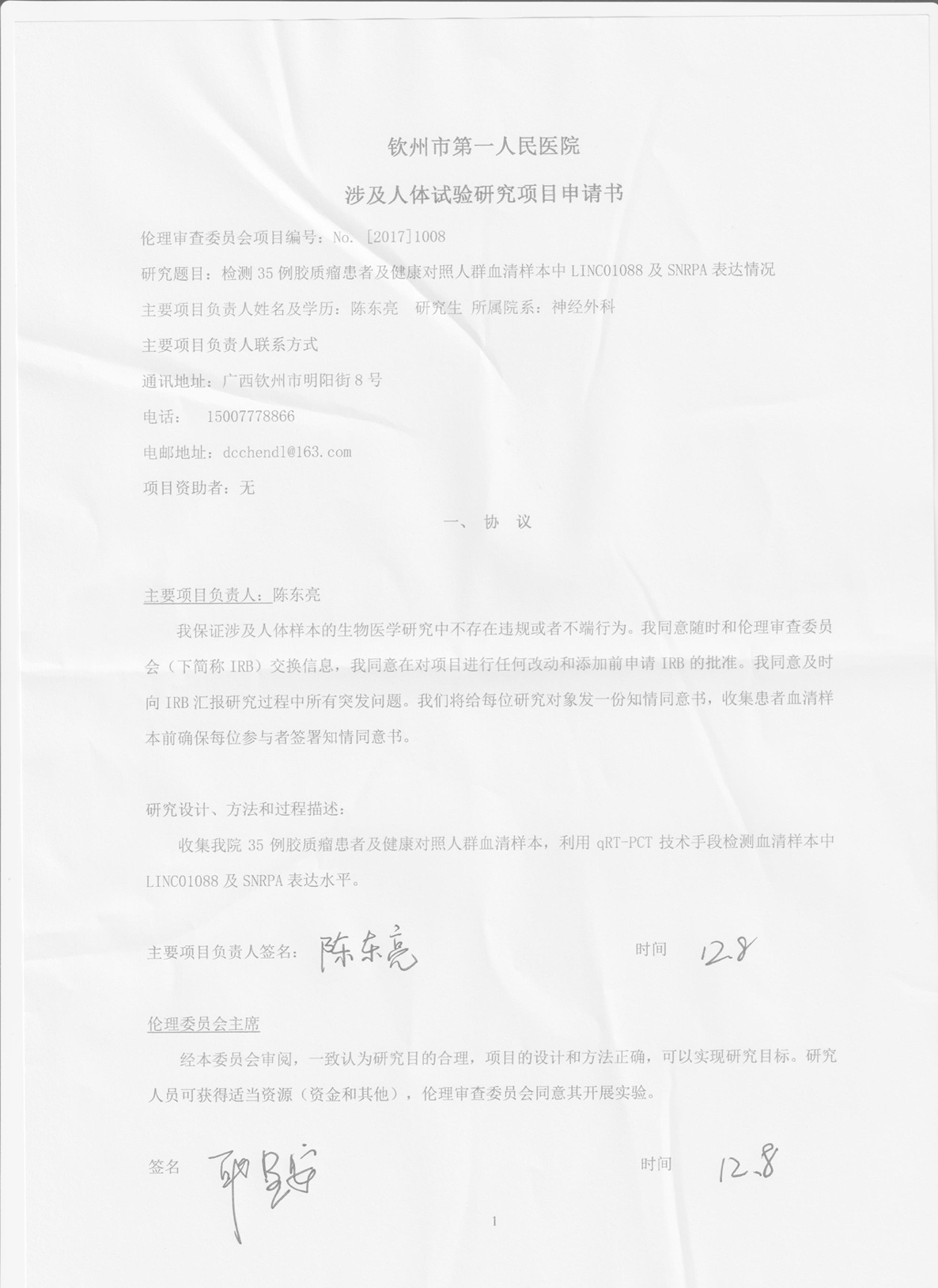

Supplement: Supplemental Material [file KBIE_A_2051786_SM4469.docx]
